# New York County Veterinary Association

**Published:** 1897-11

**Authors:** Robert W. Ellis

**Affiliations:** Secretary


Secretary.

### NEW YORK COUNTY VETERINARY ASSOCIATION.

THE regular monthly meeting of the Association was called to order at 8 o'clock P.M., at the Academy of Medicine, on Wednesday, October 6th, with the President, Dr. Huidekoper, in the chair.

The following members responded to roll-call: Drs. J. S. Cattanach, Delaney, Dair, Ellis, Farley, Foy, Gill, Huidekoper, Hanson, MacLean and MacKellar. The minutes of the previous meeting were read and approved.

The Board of Censors and Judiciary Committee had no reports to make. The Committee on Publication reported having considerable of the material of the *Blue Book* now in the hands of the printer, and the rest ready outside for corrections; also that considerable addition to the original undertaking had been considered advisable and had been accomplished, taking in every State in the Union, and the laws relative to contagious diseases in the United States and Canada.

Under the head of new business, a motion was made by Dr. Gill, and seconded by Dr. Hanson, that the President appoint a committee to draft suitable resolutions upon the death of our late member, Edward Loomes; carried. The President thereupon appointed to act on said committee Drs. Gill, Cattanach, and Farley, and directed that a recess be taken for the preparation of the resolutions. On returning, the committee submitted the following:

WHEREAS, It has pleased Almighty God to take from among us our respected professional brother, Dr. Edward Loomes, a charter-member of this Association. He was a man of great personal attraction, and his ability as a veterinarian commanded a large and remunerative practice; and by his death this Association loses a valuable member and the profession a worthy colleague; therefore, be it

*Resolved*, That we, the members of the Veterinary Medical Association of New York County, tender to his bereaved widow, in this the hour of her affliction, our heartfelt sympathy; and be it further

*Resolved*, That a copy of these resolutions be properly engrossed and presented to Mrs. Loomes.

Moved and seconded that the resolutions offered by the committee be adopted; carried. Moved and seconded that the meeting adjourn; carried.

ROBERT W. ELLIS,
Secretary.

